# Outcomes of Bilateral Deep Brain Stimulation After Magnetic Resonance‑Guided Focused Ultrasound Thalamotomy: A Case Series

**DOI:** 10.1227/neuprac.0000000000000255

**Published:** 2026-06-11

**Authors:** Andrea C. Zoana, Mitesh P. Lotia, Nigam A. Reddy, Anwar Ahmed, Chandan G. Reddy

**Affiliations:** 1 AdventHealth Neuroscience Institute, Orlando, Florida, USA;; 2 Department of Neurology, AdventHealth Innovation Tower, Orlando, Florida, USA;; 3 Department of Neurosurgery, AdventHealth Celebration, Celebration, Florida, USA;; 4 Orlando Neurosurgery, Orlando, Florida, USA

**Keywords:** Case series, Deep brain stimulation, Essential tremor, Movement disorders, MR-guided focused ultrasound, Stereotactic and functional, Thalamotomy

## Abstract

**BACKGROUND AND OBJECTIVES:**

Despite its efficacy in treating medically refractory movement disorders, magnetic resonance‑guided focused ultrasound (MRgFUS) thalamotomy may result in tremor recurrence, requiring additional interventions. The aim of this study was to evaluate the safety and efficacy of subsequent deep brain stimulation (DBS) as a management strategy for patients with recurrent or residual symptoms after MRgFUS thalamotomy.

**METHODS:**

We retrospectively reviewed 8 patients who underwent bilateral DBS after unilateral MRgFUS thalamotomy for medically refractory essential tremor (n = 7) or tremor-dominant Parkinson disease (n = 1) between January 2021 and August 2025. During this time frame, we performed 529 MRgFUS thalamotomies in 461 patients and 108 DBS procedures in 100 patients. We analyzed clinical indications, complications, and functional outcomes using the Fahn-Tolosa-Marin (FTM) Tremor Rating Scale and the Unified Parkinson's Disease Rating Scale Part III.

**RESULTS:**

The mean time from MRgFUS to bilateral DBS was 19.2 months (range: 6-48 months). Indications for DBS included tremor recurrence (n = 4), technical inability to complete MRgFUS ablation because of anatomical limitation (n = 2), and progression of contralateral symptoms (n = 2). All patients tolerated the DBS procedure well. Postoperative functional outcomes were favorable, with FTM scores in available cases improving from a mean pre-DBS score of 52.3 to 15.3 (mean FTM reduction of 70.7%). The patient with tremor-dominant Parkinson disease experienced a reduction of 64.9% in the Unified Parkinson's Disease Rating Scale Part III OFF score, improving from 57 before DBS to 20.

**CONCLUSION:**

Prior unilateral MRgFUS thalamotomy does not limit the safety and effectiveness of subsequent bilateral DBS. DBS serves as a viable treatment option in case of suboptimal MRgFUS outcomes, providing adjustable therapy and a flexible approach for managing progressive disease. In some patients, the residual MRgFUS lesion may enhance DBS efficacy, allowing adequate tremor control at lower ipsilateral stimulation thresholds.

**ABBREVIATIONS:**
ET: essential tremor
FTM: Fahn-Tolosa-Marin
HLD: hyperlipidemia
HTN: hypertension
MER: microelectrode recording
MRgFUS: magnetic resonance‑guided focused ultrasound
PD: Parkinson's disease
SDR: skull density ratio
TDPD: tremor-dominant Parkinson's disease
UPDRS: Unified Parkinson's Disease Rating Scale
VIM: ventral intermediate nucleus
VOP: ventralis oralis posterior.

Essential tremor (ET) and Parkinson disease (PD) represent the principal and most prevalent movement disorders, being responsible for most patients seeking tremor treatment. As defined by the International Parkinson and Movement Disorder Society guidelines, ET manifests as an action tremor syndrome, while PD is characterized by bradykinesia associated with resting tremor, rigidity, or both.^1,2^ The heterogeneity of PD is well established, encompassing a tremor-dominant subtype characterized by a variably reduced response to dopaminergic agents, which may lead to treatment resistance in some patients.^3^

Pharmacotherapy remains the first-line therapeutic option. However, medically refractory cases may often require further ablative or surgical intervention, such as magnetic resonance‑guided focused ultrasound (MRgFUS) thalamotomy or deep brain stimulation (DBS).

MRgFUS thalamotomy has emerged as a noninvasive treatment option,^4^ its efficacy and safety being established in the pivotal randomized controlled trial by Elias et al^5^ and further supported by long-term follow-up data.^6-10^ However, multiple reports have raised concerns regarding tremor recurrence,^11-13^ highlighting the variability of the MRgFUS ablative lesion in terms of long-term durability. Although the exact mechanisms remain unknown, recurrence is believed to relate to the duality of the underlying tremor network pathways, as a single lesion may not fully disconnect all contributing fibers.^12^

Prior studies have demonstrated successful outcomes of bilateral DBS in patients with recurrent ET^11,13^ and tremor-dominant Parkinson's disease (TDPD)^12^ tremor after MRgFUS, the largest series comprising 3 patients. Even so, data regarding the feasibility and safety of DBS as a salvage option in case of suboptimal MRgFUS outcomes remains limited.

We present a case series of 8 patients who underwent bilateral DBS after prior MRgFUS thalamotomy.

## METHODS

This is a retrospective, single-center case series of 8 patients who underwent bilateral DBS after prior unilateral MRgFUS thalamotomy for medically refractory ET or TDPD between January 2021 and August 2025. Inclusion criteria required a primary diagnosis of ET or TDPD; documented evidence of prior unilateral MRgFUS; and subsequent placement of bilateral DBS leads for recurrent, refractory, or contralateral tremor. Owing to the retrospective nature of this study, a formal written informed consent waiver was obtained from the institutional review board under the Health Insurance Portability and Accountability Act waiver of authorization, and the study was conducted in accordance with the Declaration of Helsinki and approved by the Institutional Review Board of AdventHealth. Patient demographics, comorbidities, indications, and clinical outcomes are summarized in Table 1.

**TABLE 1.** Bilateral DBS Outcomes in Patients After Unilateral MRgFUS Thalamotomy

Case	Age/sex	Indication for DBS	MRgFUS-treated side	Time to DBS (mo)	Pre-DBS functional score	Post-DBS functional score	Complications
1	67/F	Contralateral tremor progression	Left VIM	14	FTM 39	FTM 6	Transient hypoglycemia
2	65/M	Recurrence (5 mo)	Left VIM	16	FTM 37	Not recorded Subjective improvement (60%-70%)	None
3	71/M	Recurrence (3-4 mo)	Left VIM	22	UPDRS III 57 OFF	UPDRS III 20 OFF	None
4	77/F	Technical failure (insufficient heating SDR 0.47)	Right VIM	6	FTM 54	FTM 25	Small asymptomatic Cortical hemorrhage
5	81/M	Recurrence (3 mo)	Left VIM	7	FTM 61	Short-term follow-up (3 mo)	N/A
6	73/M	Anatomical targeting failure (asymmetry)	Right VIM	20	No data	Short-term follow-up (6 mo)	Transient Sinusal pause
7	70/M	Contralateral tremor progression	Left VIM	21	FTM 23	Stable postexplant	Hardware infection leading to explant
8	76/M	Recurrence (6-7 mo)	Left VIM	48	FTM 64	FTM 15	None

DBS, deep brain stimulation; F, female; FTM, Fahn-Tolosa-Marin; M, male; MRgFUS, magnetic resonance‑guided focused ultrasound; N/A, not available; SDR, skull density ratio; UPDRS, Unified Parkinson's Disease Rating Scale; VIM, ventral intermediate nucleus.

This table summarizes the clinical characteristics, history and comorbidities, indications for transition from MRgFUS to DBS, time interval between procedures, functional outcomes, and observed complications for the 8 patients in the case series.

**FIGURE 1.**
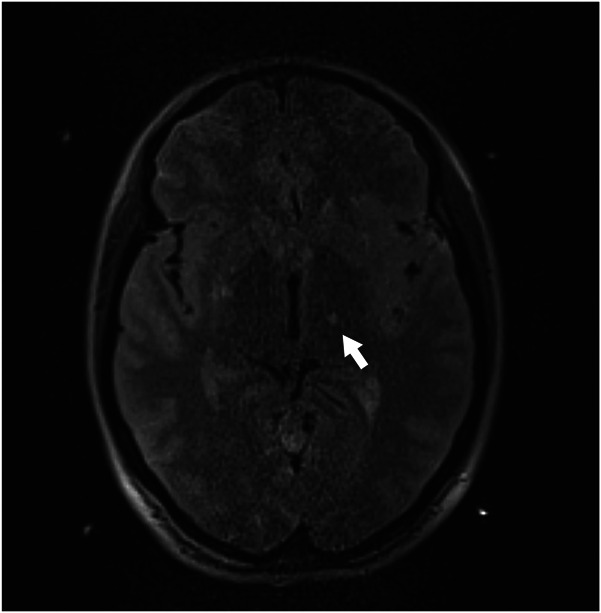
Immediate post‑magnetic resonance‑guided focused ultrasound T2-weighted constructive interference in steady state MRI showing a successful thalamotomy lesion. A typical hyperintense lesion is visible in the ventral intermediate nucleus of the thalamus (white arrow, on the patient's left), confirming acute tissue necrosis and immediate radiographic confirmation of the unilateral ablation procedure.

All patients underwent initial unilateral MRgFUS thalamotomy targeting the ventral intermediate nucleus (VIM) using the InSightec Exablate Neuro System. A T1 fast low angle shot sequence was used for target planning, and a T2-weighted constructive interference in steady state sequence was used to evaluate the lesion after treatment (Figure 1). The treatment target was heated through a series of sonication cycles and gradual temperature adjustment, aiming for a maximal temperature of 56 to 57°C. Temperature measurements during the treatment were obtained using multiecho proton resonance frequency thermometry sequences. Spiral drawing tests and quick neurologic examinations were performed before, during, and after the MRgFUS procedure to monitor tremor reduction and possible side effects.

**FIGURE 2.**
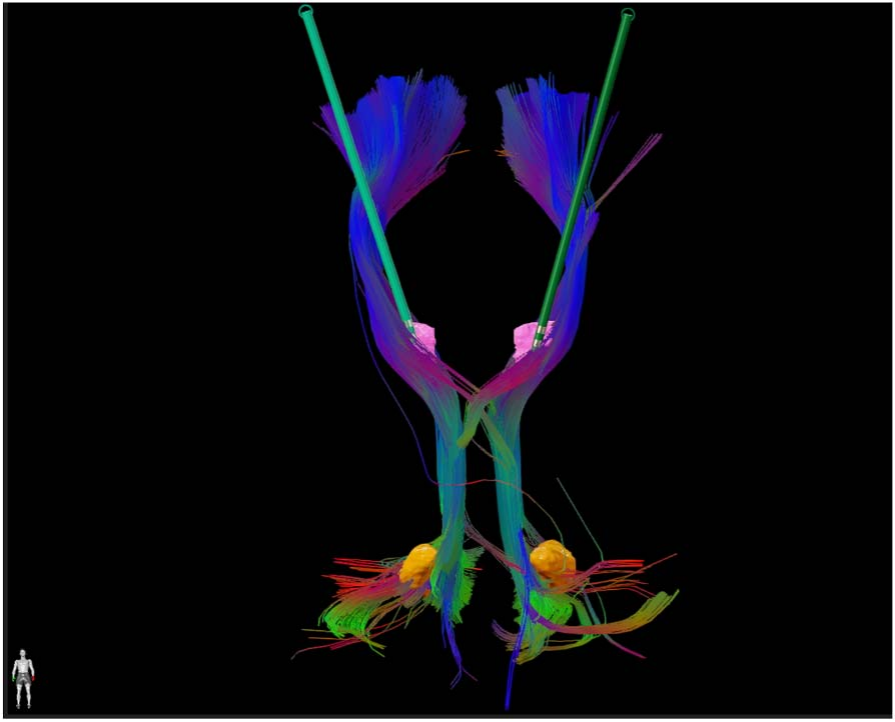
Diffusion tensor imaging tractography showing the DRTT fibers in Case 1. Brainlab images demonstrate the bilateral, interconnected nature of the tremor network, highlighting the decussating (magenta) and nondecussating portions of the DRTT segment (turquoise), ventral intermediate nucleus nucleus of the thalamus (pink), and deep brain stimulation electrode (green). DRTT is a potential therapeutic target in cases of symptom progression or tremor recurrence after unilateral ablation. DRTT, dentorubrothalamic tract.

All DBS procedures were performed under local anesthesia with sedation. Preoperative T1 with contrast, T2-weighted, T2 Fluid-Attenuated Inversion Recovery and Fast Gray Matter Acquisition T1 Inversion Recovery MRI sequences were obtained and fused with a stereotactic computed tomography scan using the BrainLAB planning system (Figure 2). The posterior-commissural point was used as the coordinate origin for all subsequent planning. Leksell G Frame was used for head stabilization, and lead insertion was performed using the Globus ExcelsiusGPS robotic navigation platform (King of Prussia, PA, USA).

**FIGURE 3.**
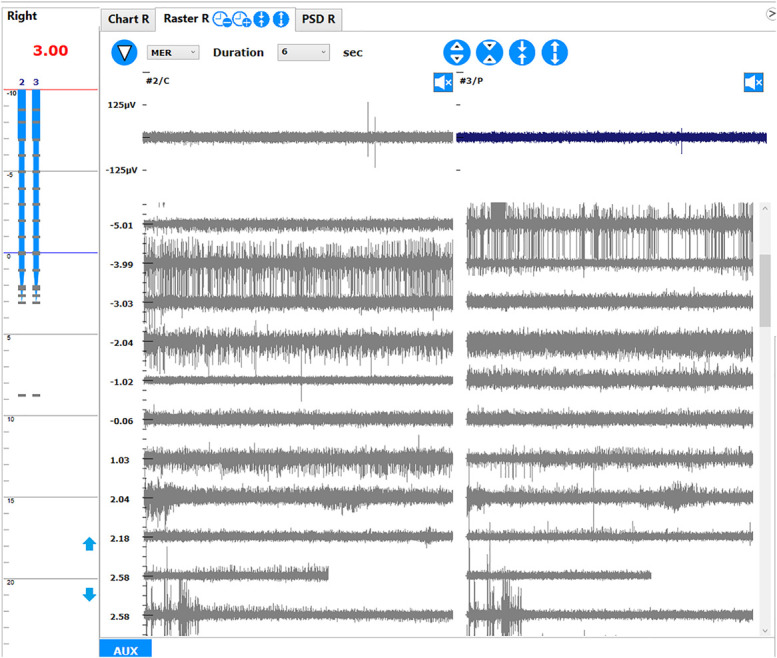
Intraoperative MER in the thalamus. MER confirms the precise anatomical and physiologic location of the ventral intermediate nucleus target, crucial for optimal lead placement. The traces show neuronal activity along the trajectory, with variations in spiking pattern indicating distinct functional regions. In some cases of prior thalamotomy, silent regions were noted during MER recording, corresponding to physiologic ablation. In such cases, deep brain stimulation leads were targeted to more anatomically active locations, more anterior to the standard ventral intermediate nucleus target, more consistent with ventralis oralis anterior/ventralis oralis posterior. AUX, auxilliary; MER, microelectrode recording; PSD R, power spectral density of right side.

Microelectrode recording (MER) and macrostimulation testing in the awake state were used to confirm target location and optimize lead placement (Figure 3). Both center and posterior tracts were tested in all patients, with subsequent final tracts varying for each case based on MER data and clinical testing. Directional DBS leads were used in all patients (Abbot Medical Infinity DBS System) using the following targets: VIM (Cases 1, 2, and 4-8), ventralis oralis posterior (VOP, Case 7, left side), and the subthalamic nucleus (Case 3).

The primary outcome measure was the change in functional tremor rating scales between the preoperative baseline and the final available clinical follow-up. The standard postoperative follow-up protocol consisted of outpatient clinical evaluations at 3, 6, and 12 months and annually thereafter, performed by the neurosurgery and neurology teams. Tremor severity was quantified using the Fahn-Tolosa-Marin (FTM) Tremor Rating Scale for patients with ET, while parkinsonian motor symptoms were quantified using the Movement Disorder Society-Unified Parkinson's Disease Rating Scale (UPDRS) Part III. Safety was assessed by documenting all intraoperative and postoperative complications. Data analysis was descriptive, focusing on mean percentage changes in tremor scores for available cases. This case series has been reported in line with the PROCESS guidelines.^14^

## RESULTS

### Cohort Characteristics and Transition Indications

A total of 8 patients (6 male, 2 female) underwent bilateral DBS after unilateral MRgFUS thalamotomy, M:F ratios being 3:1. All had MRgFUS thalamotomy of the dominant hand, with most right-hand dominant except for Cases 4 and 6 (left-hand dominant). The mean age at the time of the DBS procedure was 72.5 years (range: 65-81 years), and the average time interval between the initial MRgFUS procedure and bilateral DBS was 19.2 months (range: 6-48 months).

Indications for transitioning to bilateral DBS were tremor recurrence at 3 or more months after the MRgFUS procedure (Cases 2, 3, 5, and 8), progression of contralateral symptoms after an initially successful MRgFUS procedure (Cases 1 and 7), and technical inability to complete the ablation because of an anatomical limitation, such as suboptimal energy transmission due to frontal hyperostosis despite acceptable skull density ratio (SDR = 0.47, Case 4) and unrecognized plagiocephaly (Case 6).

Owing to limited long-term data on effectiveness of MRgFUS thalamotomy beyond 5 years, bilateral VIM DBS was performed, even in those cases with effective tremor relief after unilateral thalamotomy (Cases 1 and 7). In Case 1, the implanted lead, despite being effectively programmed, was generally turned off because it was not required to control tremor. Case 7 was explanted, but before explantation, had very low setting requirements on the previously lesioned side. MER provided useful recordings in all cases, and in Case 5, because of silent MER runs during the previously lesioned site, VOP, a slightly anterior target, was taken with good recordings and efficacy in tremor control.

Two patients were lesioned at outside institutions, prior to presenting to our center: Case 6, the patient with likely unrecognized plagiocephaly, and Case 3, the patient with TDPD.

### Safety and Complications

Overall, the procedure was tolerated well, with no permanent neurologic deficits recorded. Complications included a transient symptomatic hypoglycemic episode (Case 1), a small cortical and subdural hemorrhage (Case 4), and a 5-second episode of sinusal pause (Case 6). All patients were clinically monitored, stabilized, and cleared for discharge by the next day. One patient underwent delayed device explantation for *Staphylococcus aureus* infection (Case 7). All patient characteristics, indications, and complications are listed in Table 1.

### Outcome Measures

Four patients had available functional tremor scores (3 ET, 1 TDPD). The mean pre-DBS FTM score was 52.3 (range: 39-64). By the last follow-up visit (range: 3-6 months), the mean FTM score improved to 15.3 (range: 6-25), demonstrating a tremor reduction of 70.7%. The best outcomes were observed in Cases 1 (FTM 39-6) and 8 (FTM 64-15). The patient with TDPD (Case 3) showed significant improvement as well, with his pre-DBS UPDRS Part III OFF score of 57 improving to 25 by 6 months and to 20 by his 1-year follow-up (64.9% tremor reduction). DBS programming settings are listed in Table 2, generally in the lower range, corresponding to effectively placed leads. MRgFUS and DBS targets are listed in Table 3.

**TABLE 2.** DBS Settings for Each Patient at Last Programming

DBS programming						
Case	Left side			Right side		
	Target	Contacts	Settings	Target	Contacts	Settings
1	VIM	C+,4-	0 mA (OFF) PW 50 130 Hz	VIM	9+,11ABC-	1.70 mA PW 50 160 Hz
2	VIM	C+,3A-	2.0 mA PW 60 130 Hz	VIM	C+,10ABC-	3.6 mA PW 90 150 Hz
3	STN	C+,3AB-	2.4 mA PW 50 170 Hz	STN	C+,11AB-	2.9 mA PW 50 170 Hz
4	VIM	0+,1C-2C-	3.1 mA PW 40 160 Hz	VIM	9+,10A-	3 mA PW 50 160 Hz
5	VOP	C+,3C-	2.3 mA PW 30 154 Hz	VIM	C+,11B-	2.4 mA PW 30 160 Hz
6	VIM	2C+,3C-4-	0.8 mA PW 20 150 Hz	VIM	10+,11A-12-	1.1 mA PW 20 160 Hz
7	VIM	C+,4-	1.0 mA PW 40 130 Hz	VIM	C+,11AB-	2.7 mA PW 50 174 Hz
8	VIM	C+,2A-	3.0 mA PW 60 130 Hz	VIM	C+,10A-	1.5 mA PW 60 130 Hz

DBS, deep brain stimulation; PW, pulse width; STN, subthalamic nucleus; VIM, ventral intermediate nucleus; VOP, ventralis oralis posterior.

Targets with corresponding contacts on the Abbott directional leads (anodes and cathodes) with stimulation amplitudes, pulse width in µsec, and frequency in Hz.

**TABLE 3.** MRgFUS Parameters and DBS Targeting

Case	MRgFUS targeting								DBS targeting							
									Left				Right			
	Target VIM	ML	AP (rel to PC)	SI	SDR	Dose (kJ)	TAvg (°C)	Lesion	Target	Lat	AP (rel to PC)	SI	Target	Lat	AP	SI
1	L	−14.0	6.4	2.0	0.50	7.8	58	Y	VIM	−12.5	5.6	2	VIM	12.5	5.6	2.0
2	L	−14.0	7.4	2.0	0.65	6.0	58	Y	VIM	−13.0	9.0	0	VIM	15.0	6.4	0.8
3	L	Treated outside				9.5	60	Y	STN	−11.6	10.3	−4.5	STN	11.6	10.3	−4.7
4	R	14.0	6.7	2.0	0.47	56	46	N—frontal hyperostosis	VIM	−14.4	8.5	1	VIM	14.7	4.5	1.4
5	L	−15.8	7.2	2.0	0.70	8.0	58	Y	VOP	−15.0	6.8	0	VIM	15.5	5.3	0.7
6	R	Treated outside			0.48	21	N/A	Y	VIM	−14.0	8.9	0	VIM	14.0	8.0	0
7	L	−14.4	7.1	2.0	0.46	20	57	Y	VIM	−12.6	−7.4	1	VIM	14.0	6.2	0
8	L	−14.0	6.7	2.0	0.42	52	54	Y	VIM	−13.9	6.9	0	VIM	14.3	8.2	0.1

AP, anterior posterior; DBS, deep brain stimulation; L, left; ML, medial-lateral; MRgFUS, magnetic resonance‑guided focused ultrasound; N, no; N/A, not available; PC, posterior commissure; R, right; SDR, skull density ratio; SI, superior-inferior; STN, subthalamic nucleus; TAvg, average temperature (°C); VIM, ventral intermediate nucleus; VOP, ventralis oralis posterior; Y, yes.

MRgFUS targeting parameters, including coordinates in millimeters (ML, AP, and SI) relative to PC, with SDR, lesional energy dose in kJ, average lesional temperature at last sonication in Celsius, and presence of lesion noted clinically by tremor control initially and lasting for at least 3 months or noted on MRI. DBS targeting coordinates for left and right sides, with laterality, AP, and SI. Generally, the DBS VIM coordinates are slightly medial and inferior relative to the MRgFUS lesioning coordinate.

### Case Descriptions

#### Case 1

The patient was a 67-year-old right-hand‑dominant woman with a history of medically refractory ET and cervical radiculopathy (Figure 4). She underwent successful left-VIM MRgFUS for her disabling right-hand tremor with great long-term control. Left-hand tremor persisted, and she was referred to the DBS committee for bilateral VIM DBS evaluation, which she underwent 14 months later. The postoperative course was complicated by an episode of transient symptomatic hypoglycemia due to a preexisting recurrent endocrine disorder. By her 6-month follow-up, the FTM score improved to 6 from the pre-DBS score of 39, and control was achieved with unilateral, right-sided stimulation only, the left lead being turned off. Subsequently, her battery was changed to rechargeable without complication and with good ongoing tremor control.

FIGURE 4.Three sets of spirals from a single patient (Case 1) across 3 different time points (beforeMRgFUS, after MRgFUS but before DBS, and after DBS), with the left hand shown in the left column and the right hand in the right column. First row (**A**, **B**) spirals were drawn before any treatment. Second row (**C**, **D**) shows spirals taken 14 months after MRgFUS treatment of right-hand tremor. Third row (**E**, **F**) reflects spirals taken 6 months after bilateral DBS treatment. DBS, deep brain stimulation; MRgFUS, magnetic resonance‑guided focused ultrasound.

**Figure:**
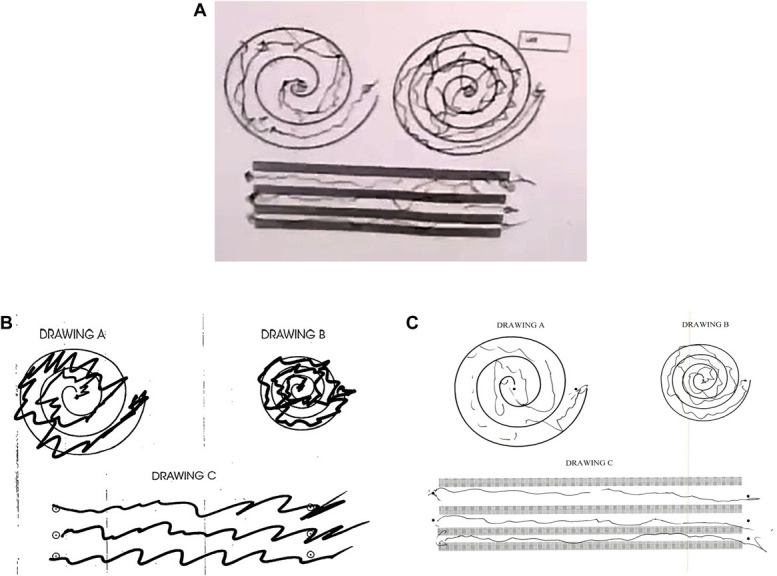

**Figure:**
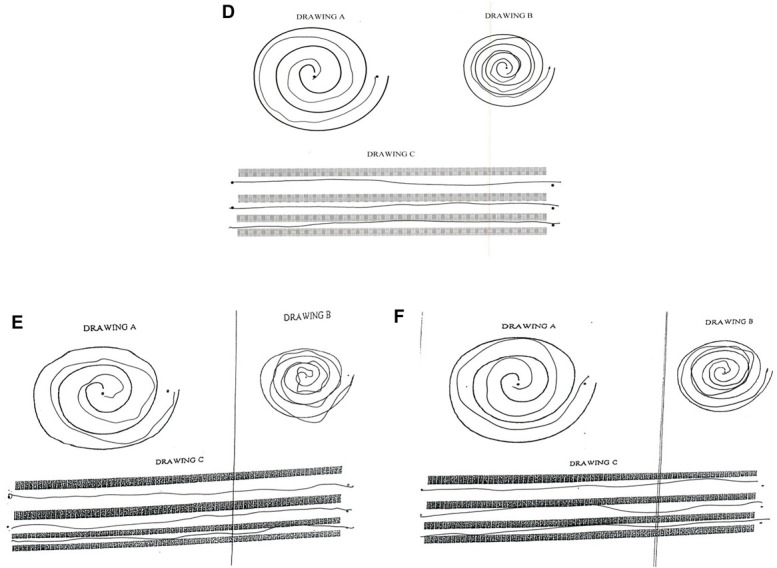

#### Case 2

The patient was a 65-year-old right-hand‑dominant man with a history of medically refractory ET, hypertension (HTN), hyperlipidemia (HLD), and type 1 diabetes who underwent left-VIM MRgFUS for his right-hand tremor. Although initially effective, tremor control faded in approximately 5 months. Bilateral VIM DBS was performed 16 months after the MRgFUS procedure, and the patient reported a subjective improvement of 60% to 70% by his second programming. At the 3-month follow-up, he reported worsening of left-hand tremor after carpal tunnel surgery, with residual left thumb tremor. He underwent botulinum toxin injection with no benefit. His dopamine transporter scan was normal, excluding the possibility of underlying PD, and his right-hand tremor remained overall well controlled.

#### Case 3

The patient was a 71-year-old right-hand‑dominant man with a history of medically refractory TDPD (dopamine transporter scan positive for presynaptic striatal dopaminergic deficit), HTN, hypercholesterolemia, severe obesity (body mass index >40), and obstructive sleep apnea (treated with continuous positive airway pressure). At an outside institution, he underwent left-VIM MRgFUS for his right-hand tremor. Although initially successful, tremor recurred around 3 to 4 months later, and he was referred to the DBS committee for bilateral subthalamic nucleus DBS evaluation. DBS was performed 22 months after the MRgFUS procedure with no periprocedural or postoperative complications. His motor function improved significantly, with an UPDRS Part III OFF score improvement from 57 before DBS to 25 at 6 months and 20 by his 1-year follow-up.

#### Case 4

The patient was a 77-year-old left-hand‑dominant woman with a history of medically refractory ET, HTN, atrial fibrillation, diabetes, and cervical dystonia who underwent right-VIM MRgFUS for her left-hand tremor. The procedure was ineffective because of suboptimal energy transmission secondary to frontal hyperostosis. Despite adequate SDR (=0.47), energy delivery (56 kJ), and sufficient number of elements (992 of 1024), her brain only heated to 46°C, unable to achieve the ablative temperature threshold. She was referred to the DBS committee for bilateral VIM DBS evaluation, which was performed 6 months after the MRgFUS procedure. At the 6-month follow-up, her FTM score improved to 25 from 54 before DBS. Her follow-up was complicated by a fall associated with alcohol consumption, yet the patient denied worsening of tremors and there was no evidence of damage to the DBS system.

#### Case 5

The patient was an 81-year-old right-hand‑dominant man with a history of medically refractory ET, restless leg syndrome, HTN, and HLD who experienced recurrence of right-hand tremor and severe contralateral tremor progression around 6 months after the MRgFUS procedure. Although present on the immediate repeat MRI, the MRgFUS lesion was not visible on a later follow-up MRI. He was deemed an excellent candidate for bilateral DBS and underwent the procedure 7 months after MRgFUS, with the left VOP and right VIM as final targets. The patient had short-term follow-up of 3 months, with effective stimulation at low amplitudes, and then was subsequently incarcerated and lost to follow-up.

#### Case 6

**FIGURE 5.**
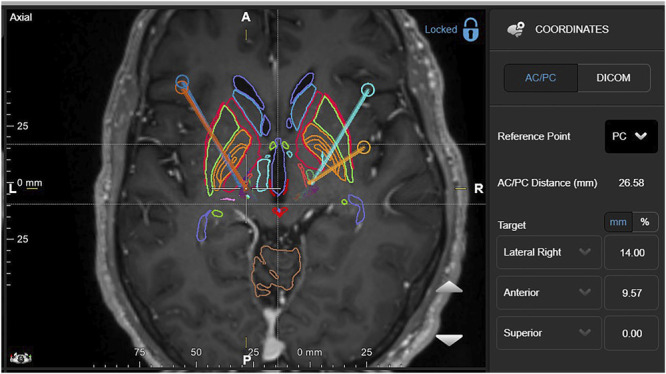
Brainlab preoperative planning image for Case 6, demonstrating anatomical asymmetry. This axial view shows a significant anterior shift of the right ventral intermediate nucleus target by 9.57 mm, approximately 3 mm anterior to the standard position. This anatomical finding is a possible contributing factor to the technical failure of the initial magnetic resonance‑guided focused ultrasound procedure and highlights the advantage of precision deep brain stimulation targeting in cases of plagiocephaly or variant anatomy. AC, anterior commissure; DICOM, digital imaging and communications in medicine; PC, posterior commissure.

The patient was a 73-year-old left-hand‑dominant man with a history of medically refractory ET, HTN, and HLD, who experienced recurrence of left-hand tremor by 1 month after the MRgFUS procedure (right VIM), performed at an outside institution. Subsequent imaging revealed an anatomical right-VIM shift, suggesting a possibility of brain asymmetry (Figure 5), consistent with plagiocephaly. After DBS committee evaluation, the patient underwent bilateral VIM DBS, performed around 20 months after the MRgFUS procedure. Postoperatively, he experienced an episode of transient sinusal pause associated with severe headache and no syncopal symptoms. He was clinically monitored and cleared up for discharge the next day. The patient had a short-term follow-up at 6 months, with different task-specific programs, pleased with his DBS outcome.

#### Case 7

The patient was a 70-year-old right-hand‑dominant man with a history of medically refractory ET, HTN, HLD, obesity (body mass index = 39.2), and obstructive sleep apnea who underwent successful left-VIM MRgFUS for his right-hand tremor. He was referred to the DBS committee for bilateral VIM DBS evaluation after the progression of his contralateral tremor and underwent the procedure 21 months after MRgFUS. At around 3 months after DBS, he came back to the clinic after noticing some drainage around the right incision site. Clinical examination showed wound dehiscence with a large scab and serosanguinous drainage, raising concern for hardware infection. Cultures returned positive for *S. aureus*, and the patient underwent device explantation. Despite this complication, his tremor remained stable compared with the preoperative state, with the right hand showing better control than the left, an outcome attributed to the possible durable effect of the initial MRgFUS lesion.

#### Case 8

The patient was a 76-year-old right-hand‑dominant man with a history of medically refractory ET, HTN, atrial fibrillation, coronary artery disease, and skin cancer who experienced recurrence of his right-hand tremor at 6 to 7 months after MRgFUS. He was considered a poor candidate for repeat MRgFUS because of low SDR (=0.42), requiring 52 kJ to achieve 54°C, being referred to the DBS committee for bilateral VIM DBS evaluation, which was performed 48 months later. By the 3-month follow-up, his FTM score improved significantly from 64 before DBS to 15, with very good tremor control noted.

## DISCUSSION

Tremor recurrence after unilateral MRgFUS thalamotomy is a known phenomenon in a small subset of patients.^6^ Management in this challenging scenario remains an open question: Should one consider a repeat MRgFUS lesioning procedure or transition to an alternative, adjustable therapy such as DBS?

When considering whether to proceed with repeat MRgFUS thalamotomy or DBS, these points are considered: Was the SDR and original skull geometry favorable to lesioning? Was the lesion placed in the right spot, and was there initial effective tremor control? Was the diagnosis correct? Then, we proceed with a DBS planning MRI and make a decision together with our neurology team and the patient. Of our 8 patients, 1 could not be effectively lesioned because of frontal hyperostosis. The remaining patients had satisfactory immediate benefit from lesioning lasting at least 3 months. One patient came to us with PD, already treated by MRgFUS at an outside institution, although our preference at the current time, with the currently available targets, is to treat PD with DBS. While performing DBS, we noted that MER may sometimes, but not always, have silent physiology during a previously lesioned site, suggesting anterior moves for lead placement in such situations, as can be verified clinically by test stimulation.

During this time frame, we performed 529 MRgFUS thalamotomies in 461 patients (with 68 returning for second-side treatment) and 108 DBS procedures in 100 patients. The 8 patients reported here represent a small portion of the original set.

The possible risks of sequential contralateral MRgFUS thalamotomy may prompt consideration of DBS. Furthermore, DBS becomes the preferred option in patients with persistent side effects from the initial unilateral ablation. Our early experience was also shaped by the initial lack of approval for second-side thalamotomy (approved in December 2022). Interestingly, in some patients, the presence of a thalamotomy had a synergistic effect with the DBS lead, requiring lower settings. Owing to the limited long-term known efficacy of the MRgFUS thalamotomy, our preference was to implant bilateral VIM, even in cases of effective thalamotomy. Our case series provides compelling evidence that bilateral DBS is a technically feasible, safe, and effective salvage strategy after suboptimal MRgFUS outcomes.

### Limitations

The main limitations of this study include its retrospective, single-center design, along with the small sample size, absence of quantifiable tremor outcomes in some patients, and variable duration of follow-up. Future prospective studies are needed to define long-term patient satisfaction and compare functional outcomes between sequential therapy and primary DBS.

## CONCLUSION

Our findings reinforce that bilateral DBS is a valuable and safe salvage option for patients with recurrent, refractory, or progressed contralateral tremor after unilateral MRgFUS thalamotomy. The technical insights regarding anatomical limitations and the potential for synergistic outcomes inform both preoperative planning and surgical techniques.
